# Intratumor heterogeneity: the hidden barrier to immunotherapy against MSI tumors from the perspective of IFN-γ signaling and tumor-infiltrating lymphocytes

**DOI:** 10.1186/s13045-021-01166-3

**Published:** 2021-10-07

**Authors:** Wantao Wu, Yihan Liu, Shan Zeng, Ying Han, Hong Shen

**Affiliations:** 1 Department of Oncology, Xiangya Hospital, Central South University, Changsha, Hunan People’s Republic of China 410008; 2 Key Laboratory for Molecular Radiation Oncology of Hunan Province, Xiangya Hospital, Central South University, Changsha, Hunan People’s Republic of China 410008; 3 National Clinical Research Center for Geriatric Disorders, Xiangya Hospital, Central South University, Changsha, Hunan 410008 People’s Republic of China

**Keywords:** Microsatellite instability, Immunotherapy, Tumor-infiltrating lymphocytes, IFN-γ signaling, Heterogeneity

## Abstract

In this era of precision medicine, with the help of biomarkers, immunotherapy has significantly improved prognosis of many patients with malignant tumor. Deficient mismatch repair (dMMR)/microsatellite instability (MSI) status is used as a biomarker in clinical practice to predict favorable response to immunotherapy and prognosis. MSI is an important characteristic which facilitates mutation and improves the likelihood of a favorable response to immunotherapy. However, many patients with dMMR/MSI still respond poorly to immunotherapies, which partly results from intratumor heterogeneity propelled by dMMR/MSI. In this review, we discuss how dMMR/MSI facilitates mutations in tumor cells and generates intratumor heterogeneity, especially through type II interferon (IFN-γ) signaling and tumor-infiltrating lymphocytes (TILs). We discuss the mechanism of immunotherapy from the perspective of dMMR/MSI, molecular pathways and TILs, and we discuss how intratumor heterogeneity hinders the therapeutic effect of immunotherapy. Finally, we summarize present techniques and strategies to look at the tumor as a whole to design personalized regimes and achieve favorable prognosis.

## Background

Immunotherapies have had promising effects on many cancer patients. In order to evaluate the response to immunotherapy, deficient mismatch repair (dMMR)/microsatellite instability (MSI) status has been widely exploited by practitioners, since it is found extensively across diverse types of cancer. dMMR/MSI is associated with improved outcomes independently of other clinical prognostic factors, such as disease stage [1]. Therefore, many clinical researchers suggest that dMMR/MSI contributes to high efficacy of immunotherapy in different tumor types [2–4].

Deficient MMR system and instable genomic status led to accumulation of somatic mutations, especially frameshift mutations [2], which generate subclones with neoantigens. These neoantigens are recognized as non-self and elicit anti-tumor responses including higher tumor-infiltrating lymphocyte (TIL) grade and expression of type II interferon (IFN-γ)-related genes, such as those encoding programmed cell death 1 ligand 1 (PD-L1), cytotoxic T lymphocyte-associated antigen-4 (CTLA-4), lymphocyte activation gene-3 (LAG-3) and indolamine-2,3-dioxygenase (IDO) [5, 6]. Nevertheless, as the depth of research grows, dMMR/MSI has been regarded as a double-edged sword in immunotherapy. That is, dMMR/MSI also correlates with resistance to immunotherapy, resulting from complex mechanisms such as frequent immunoediting of WNT/β-catenin signaling, antigen presentation machinery and IFN-γ signaling [7–11].

dMMR/MSI is one of the most important drivers of intratumor heterogeneity (ITH) [12], which refers to the different states within a tumor such as genomic instability, epigenetic abnormality, acetylation, gene expression dysregulation, post-translation modifications, biological behaviors, tumor microenvironment, T cell receptor and heterogeneous response to therapies [13]. ITH is present spatially and temporally. Spatial heterogeneity is defined as distinct genetic alterations and phenotypes between tumor cells; while temporal heterogeneity is embodied in the evolvement of subclones during natural tumor progressing and therapeutic interventions. Generally, tumors start out as a heterogeneous mixture, and immune selective pressure imposed by immunotherapy facilitates outgrowth of resistant clones and elimination of sensitive ones. ITH is found in a variety of tumors and predicts prognosis of targeted therapies [14].

**Fig. 1:**
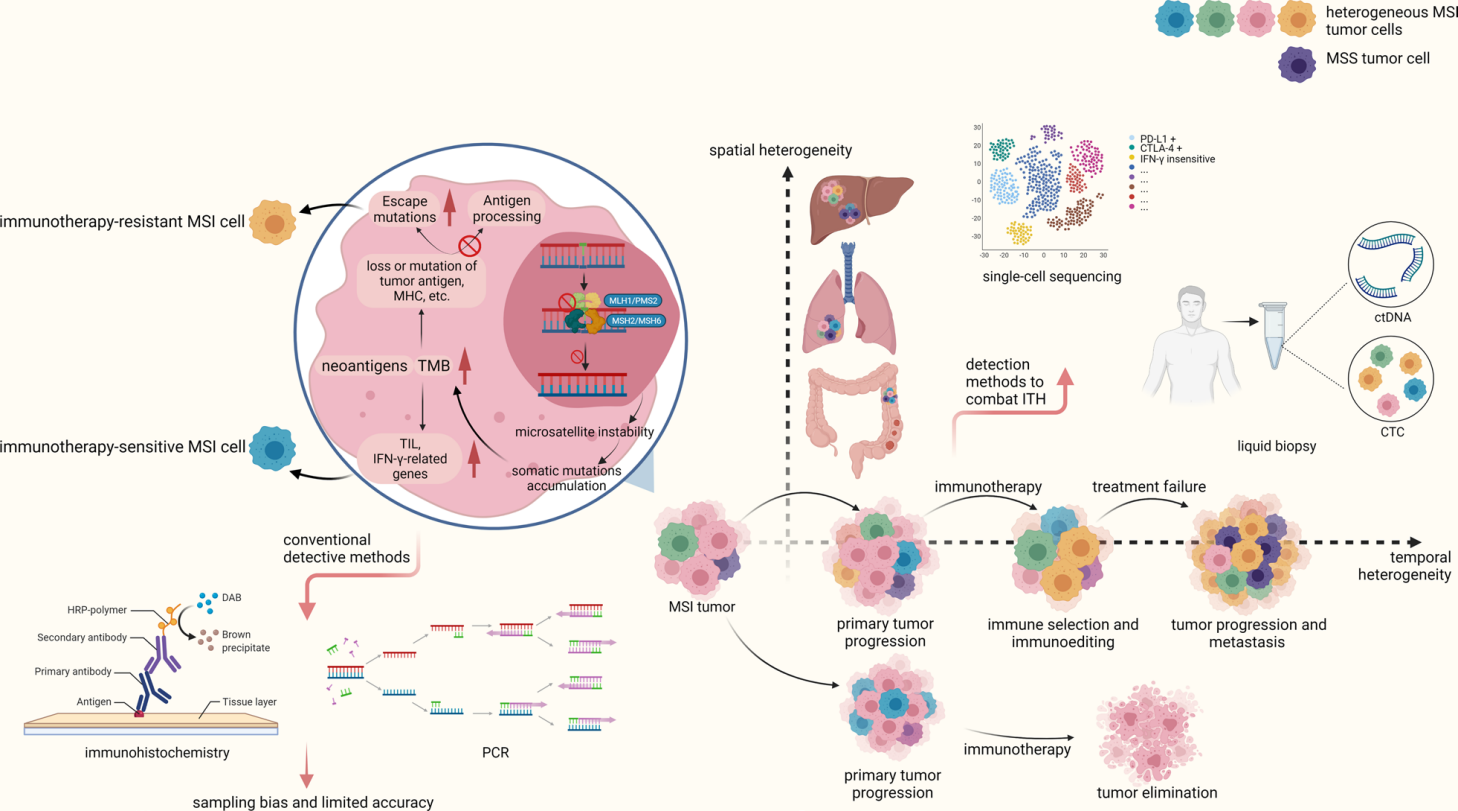
Progression of MSI tumor. In dMMR tumors, dysfunction in mismatch repair system cannot repair DNA mismatches, leading to DNA sequence alterations especially in microsatellites. With the accumulation of DNA sequence alterations, the tumor mutation burden gradually grows, and tumor cells are evolving into different subclones harboring heterogeneous neoantigens and characteristics. The application of immunotherapy eliminates many tumor cells and puts tumor under immune selection and immunoediting. Subclones which are resistant to immunotherapy grow out. Finally, the treatment-resistant primary tumor and metastases with heterogeneous subclones progress. Besides, status of MMR within a tumor is heterogeneous. MSS tumor cells may exist in dMMR/MSI tumors as well, and these cells do not respond to immunotherapy at the first place. As many of the MSI cells are eliminated, MSS tumor cells can grow out, leading to resistance to immunotherapy. Therefore, utilizing new detection methods to combat ITH is crucial to characterize tumor landscape

ITH may result in sampling bias of biomarkers in cancer immunotherapy, such as programmed cell death protein-1 (PD-1), tumor mutation burden (TMB) and dMMR/MSI, and lead to entirely different clinical consequences. In other words, the current single tumor specimen underestimates the genomic spectrum variety across the tumor [15]. Different technologies have been invented to enable simultaneous deep analysis of single cells integrating genome, epigenome and transcriptome information [16]. ITH characterization is better than ever through bulk cell profile analysis and depiction of single cells in different regions via multiomics and is shown to significantly impact the immune response and prognosis of cancer patients (Fig. 1). Studies show that increased ITH is associated with worse anti-PD-1 therapy efficacy and “biomarker-oriented heterogeneity” determines drug sensitivity of each subclone [17–19]. These phenomena may explain why prognosis for a large proportion of patients remains poor after immunotherapy treatment with the target molecule. Therefore, ITH is a huge obstacle in treating tumors effectively.

In this review, we discuss the two-sided effects of dMMR/MSI on immunotherapy. We summarize recent immunotherapy studies, including immune checkpoint blockade (ICB), adoptive cell transfer (ACT) and vaccine, and explore the effect of ITH on factors such as dMMR/MSI, TIL, IFN-γ and immune checkpoints. Due to the widespread effects of ITH in tumors [20], methods to combat spatial and temporal heterogeneity should be utilized to learn the big picture of tumor and guide therapy selection. We review the latest advances in single-cell sequencing and liquid biopsy, including circulating tumor DNA (ctDNA) and circulating tumor cells (CTC). Dynamic tumor cell profiling could translate into clinical applications for promising tumor therapy in the near future.

### MSI plays a vital role in the generation of intratumor heterogeneity

**Fig. 2:**
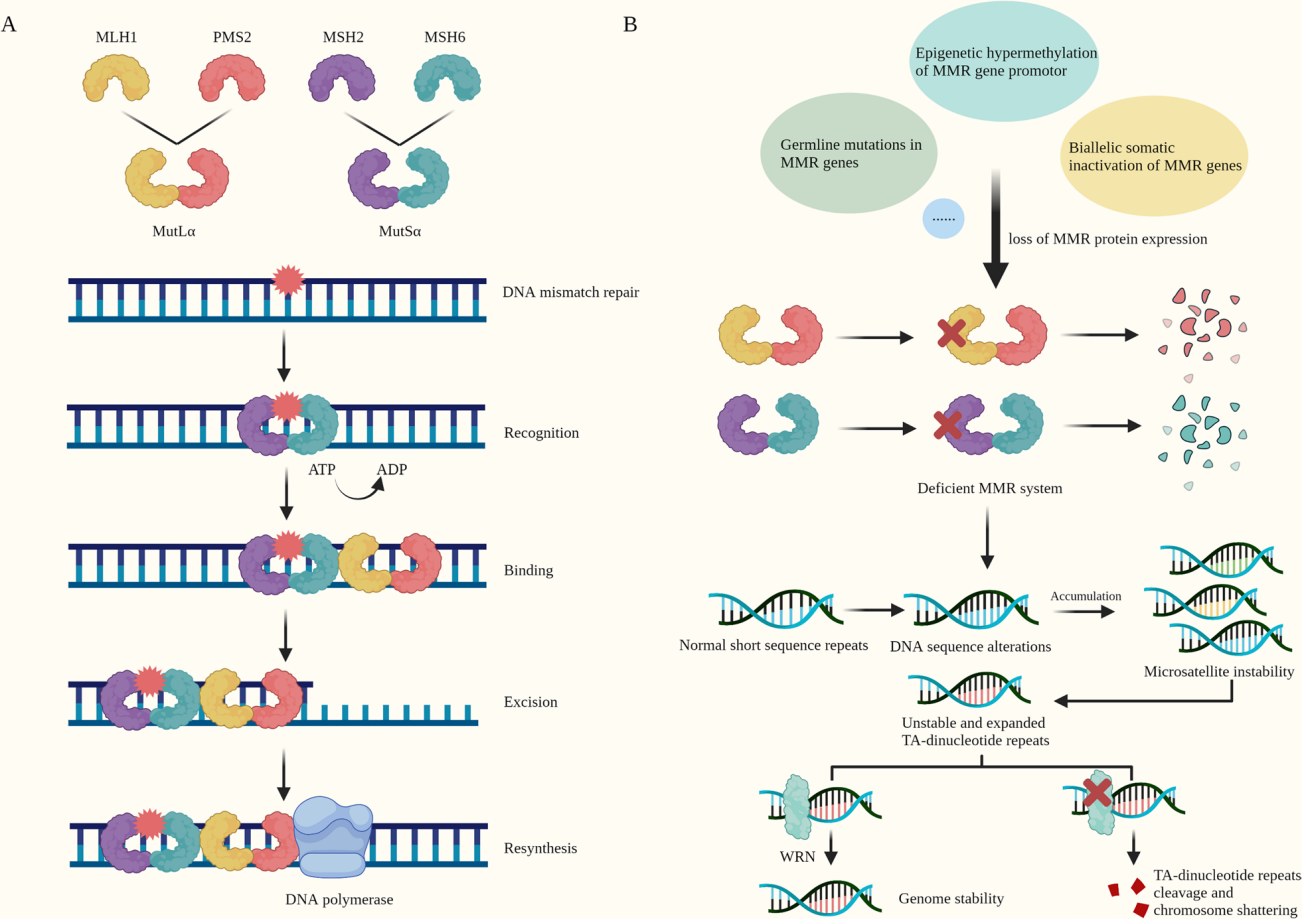
The mechanism of normal MMR system and dMMR/MSI.** a** The MMR system consists of four major proteins: MLH1, MSH2, MSH6 and PMS2. They work in the form of heterodimers: MLH1 couples with PMS2 (MutLα), and MSH2 couples with MSH6 (MutSα). MutSα recognizes DNA mismatched base errors, creates a sliding clamp around DNA, undergoes an ATP-driven conformational switch and subsequently binds MutLα. The complexes interact with enzymes including DNA polymerase to excise the mismatch and resynthesize DNA. **b** Germline mutations in MMR genes, epigenetic hypermethylation of MMR gene promotor or biallelic somatic inactivation of MMR genes could lead to loss of MMR protein expression and deficient MMR system. dMMR is likely to cause DNA sequence alterations in microsatellites, and accumulation of which is called MSI. TA-dinucleotide repeats are unstable and expanded in dMMR/MSI cells. These cells are dependent on WRN to maintain genome stability, and avoid TA-dinucleotide repeats cleavage and chromosome shattering

dMMR/MSI is generalized across different cancer types, occurring with different frequencies and signatures. It is most commonly found in colorectal, endometrial and gastric cancers, but also in ovarian, cervical and prostate cancers [21–25] (Table 1). The MMR system consists of four major proteins: MLH1, MSH2, MSH6 and PMS2, which identify and correct DNA mismatches in the form of heterodimers: MLH1 couples with PMS2, PMS1 or MLH3 (forming MutLα, MutLβ or MutLγ complexes), and MSH2 couples with MSH6 or MSH3 (forming MutSα and MutSβ complexes) [26, 27]. MutSa could recognize DNA mismatched base errors, create a sliding clamp around DNA, undergo an ATP-driven conformational switch and subsequently bind MutLα to interact with enzymes such as DNA polymerase, excise the mismatch and resynthesize DNA [27–29] (Fig. 2). Germline mutations in MMR genes, epigenetic hypermethylation of MMR gene promotor or biallelic somatic inactivation of MMR genes could lead to loss of MMR protein expression [30]. Among them, loss of MLH1 and/or PMS2 occurs at higher frequency than loss of MSH2 or MSH6, and loss of MLH1/PMS2 co-expression is more common than loss of MSH2/MSH6 co-expression [31] (Table 2). Tumors with at least one MMR protein loss by immunohistochemical (IHC) detection are called dMMR tumors, in contrast to MMR-proficient (pMMR) tumors. And generally, loss of MLH1 or MSH2 leads to degradation of PMS2 or MSH6, respectively [29]. A deficient MMR system is likely to cause DNA sequence alterations especially in microsatellites, which are short tandem repeats scattered throughout the genome. An accumulation of errors in the microsatellites is called MSI, a hypermutator phenotype associated with hereditary and sporadic tumors [27]. Based on microsatellite loci analysis, tumors with an instability of at least two loci out of BAT-25, BAT-26, D2S123, D5S346, D17S250 (Bethesda panel) or three loci out of BAT-25, BAT-26, NR-21, NR-24, NR-27 (Pentaplex panel) are considered as MSI, in contrast to microsatellite stable (MSS) [2, 28].

**Table 1:** Frequency of dMMR/MSI across tumors

Tumor type	dMMR/MSI (%)	References
CRC	19	[21]
	17	[243, 244]
	15	[25]
	8	[245]
	6	[2]
EC	33	[246]
	30	[21]
	28	[244]
	17	[2]
GC	22	[247]
	21	[244]
	15	[25]
	8	[2]
OC	12	[25]
	10	[248]
	2–3	[2, 21, 244]
Cervical cancer	2–10	[24]
	2–3	[2, 244]
Prostate cancer	< 2%	[24]
	1–2	[2, 21, 244]
HCC	16	[249]
	2–3	[2, 244]
PC	1–2	[2, 244]
GBM	1	[2, 244]
HNSCC	1	[21, 244]
RCC	2	[250]
	1–2	[21, 244]
UTUC	28.1	[251]
Lung adenocarcinoma	< 1	[2, 21]
	0	[244]
Lung squamous cell cancer	1	[21, 244]
Cholangiocarcinoma	2	[2]
Rectal cancer	9	[244]
	3	[21]
Ampullary carcinoma	10	[252]
Thyroid cancer	2	[2]
UCEC	17–31.37	[29]
ACC	4.35	[29]
ESCA	1.63	[29]
SKCM	1	[202]
	0–0.64	[29]

*CRC* Colorectal cancer, *EC* Endometrial cancer, *GC* Gastric cancer*, OC* Ovarian cancer*, HCC* Hepatocellular cancer*, PC* Pancreatic carcinoma, *GBM* Glioblastoma multiforme, *HNSCC* Head and neck squamous cell carcinoma*, RCC* Renal cell cancer, *UTUC* Upper tract urothelial carcinoma, *UCEC* uterine corpus endometrial carcinoma, *ACC* adrenocortical carcinoma, *ESCA* esophageal carcinoma, *SKCM* skin cutaneous melanoma

**Table 2:** Frequency of loss of MMR proteins across tumors

MLH1 (%)	PMS2 (%)	MSH2 (%)	MSH6 (%)	MutLα(MLH1/PMS2)(%)	MutSα(MSH2/MSH6) (%)	Tumor type	N	Reference
78.2	82	12.1	15.9	77.2	11.5	MSI solid tumors	1057	[31]
N/A	N/A	N/A	N/A	3.7	1.8	GC	107	[253]
N/A	1.8	N/A	N/A	20.4	5.3	CRC	113	[254]
N/A	N/A	5.9	11.8	41.2	N/A	Undifferentiated GIC and PC	17	[255]
30.2	34.9	55.8	46.5	N/A	N/A	High-grade gliomas	355	[256]
7.1(partially negative)	7.1(partially negative)	7.1(partially negative)	7.1(partially negative)	N/A	N/A	Primary GBM	57	[257]
7.1(partially negative)	7.1(partially negative),7.1(completely negative)	14.3(partially negative),7.1(completely negative)	57.1(partially negative),28.6(completely negative)	N/A	N/A	Recurrent GBM	57	[257]
0.9	12.3	2.7	16.8	N/A	N/A	Prostate cancer	220	[258]
59.5	67.6	18.9	32.4	N/A	N/A	Endometrial endometrioid adenocarcinoma	107	[259]
23.8	N/A	14.8	9.3	N/A	N/A	Endometrioid endometrial carcinoma	486	[260]
83.34	3.33	1.37	4.11	N/A	N/A	CRC	1000	[261]
20	13.3	33.3	33.3	N/A	N/A	CRC,EC	15	[262]
23	N/A	N/A	N/A	N/A	N/A	pNETs	48	[263]
36	N/A	16	N/A	N/A	N/A	pNETs	55	[264]

*GC* Gastric cancer*, CRC* Colorectal cancer*, GIC* Gastrointestinal cancer*, PC* Pancreatic carcinoma*, GBM* Glioblastoma multiforme*, EC* Endometrial cancer*, Pnet* Pancreatic neuroendocrine tumor, *N/A* Not applicable

BRAF V600E mutation is often associated with MLH1 promoter hypermethylation, resulting in simultaneous loss of MLH1 and PMS2, which has been reported in 70% of dMMR/MSI tumors [24, 32]. BRAF mutation is related to negative prognosis in CRC, but due to its strong association with MSI phenotype, studies found that the positive prognosis impact of MSI could alleviate or overcome the negative effect [33, 34]. Furthermore, immunotherapy combined with BRAF inhibitor has been found to benefit patients with BRAF mutation, providing additional treatment target for patients unlikely to have long-lasting response to immunotherapy alone [35]. Moreover, the latest studies found that TA-dinucleotide repeats were highly unstable in dMMR/MSI cells and underwent large-scale expansions. Werner helicase (WRN), a member of the RecQ family of DNA helicases crucial for maintaining genome stability, was important to avoid TA-dinucleotide repeats cleavage and massive chromosome shattering [36], indicating WRN as a synthetic lethal vulnerability for dMMR/MSI tumors. Indeed, the dependency of WRN was observed widespread in dMMR/MSI tumors [37]. WRN knockout could induce double-strand DNA breaks, and selectively impair the viability of dMMR/MSI cells by nuclear abnormalities and cell division defects, which might be influenced by the loss of MSH2 or MLH1 [38, 39] (Fig. 2). Due to the finding that WRN dependency was associated with resistance to immunotherapy in dMMR/MSI CRC models [40], WRN may serve as a potential target for treating dMMR/MSI tumors.

Essentially, dMMR/MSI facilitates the process of mutations in tumor cells and propels ITH, leading to the immune evasion of tumors [41, 42]. A systemic review by European Society for Medical Oncology described high percentages of concurrence of TMB-high and MSI-high in cancers such as colorectal cancers and endometrial cancers [43]. In an analysis of glioma, defects in mismatch repair (MMR) genes were found to play a vital role in the pathways to high tumor mutational burden [44]. Even though TMB has been used as a predictor for immunotherapy response, researches have noticed that tumors with equally high TMB levels presented with diverse immune response [45]. A key cause is that TMB resulted from increased genomic instability is considered the fundamental contributor of ITH [12]. In a mouse model, researchers managed to uncouple effects of ITH and TMB, and they discovered that ITH can be a predictor of immunotherapy response independent of TMB [46]. During tissue repair, inflammation and injury-induced cell turnover may inevitably lead to mutation acquisition; subsequently, mutations generated through this process are faced with natural selection pressure by the host’s immune response (Fig. 1). With the joint effort of intratumoral competition and immunoediting, this evolutionary process may result in ITH with a unique mutational composition across the lesion [47]. One study found that most mutational signatures are ubiquitous between normal colon cancer recesses and adjacent normal recesses and sporadic mutations are not significantly different either. Nevertheless, mutations in specific genes (BRAF, APC, KRAS, TP53, etc.) are more frequent in those with colon cancer [48].

Immunotherapy not only acts as a strong immune selection pressure through which subclones bearing pre-existing resistant phenotype grow out, but also generates new subclone driver events [41, 49]. This change in mutation landscape after treatment contributes to temporal intratumor heterogeneity, and temporal response and follow-up are especially important in response to treatment; while change of the subclones is bound to change in the immune response. In colorectal cancer associated with colitis, cancer cells undergo genetic mutations in the early stage of tumorigenesis [50]. In some cancer types, the driver mutations and DNA methylation level may be determined in the early stage of tumorigenesis [51, 52]. In polyclonal tumors, significant tumor heterogeneity is discovered by seeding the initiating sublineages at the early stage [42]. In some other tumors, tumor evolution in branched sublineages makes up most driver mutations of tumorigenesis [53]. No matter what evolution process the tumor takes, they present ITH. In studies covering several cancer types, ITH has been deemed as a symbol of tumor progression, as high ITH often correlates with decreased immune activity and exhausted immune microenvironment [44, 54, 55]. ITH in the expression level of IFN-γ and TILs influences the efficacy of immunotherapy. Among diverse groups of TILs, our review focuses on tumor-infiltrating T cells that are directly linked to cytotoxic effects against tumor cells and their ITH is well studied.

IFN-γ is a major member of the IFN cytokine superfamily produced by T cells and nature killer (NK) cells upon the recognition of tumor antigens. It has a wide range of biological functions such as antivirus, anti-tumor and immune regulation, through induction of multiple proteins via IFN-γ stimulated genes (ISGs). With the discovery that the expression of PD-L1 within tumors is focal and heterogeneous both spatially and temporally [47, 56, 57], other studies on ITH of IFN-γ signaling have been published in succession. In the lung adenocarcinoma (LUAD) patient-derived xenografts (PDXs), Ke-Yue Ma et al. discovered that IFN-γ signaling pathway genes were heterogenous and coregulated with other immune-related genes including PD-L1, MHCII and IDO. The downregulation of IFN-γ signaling is associated with an acquired phenotypic resistance [58].

Somatic mutations of tumor are essential for neoantigen expression and consequent immune infiltration [2, 59]. Antigen-presenting cells and TILs play an indispensable role in recognizing tumor neoantigens and generating cytotoxic effects against tumor cells. The process of neoantigen presentation and mechanisms by which tumor cells evade immune recognition have been reviewed elsewhere [60]. Among TILs, ITH of the T cell repertoire has been widely recognized, and T cell clusters bring about pivotal and direct effects on tumors, which is the focus of this review. The two-sided role of B cells and the antibody repertoire has been delineated elsewhere [61]. For patients who respond to immunotherapy, the vanished tumor neoantigen is in line with the expansion of TIL clonotypes [62]. Theoretically, the greater the mutation burden of a tumor, the stronger the provoked immune response. TMB, a biomarker reflecting the mutation degree of tumor cells, is positively linked with the prognosis of patients receiving immune checkpoint inhibitors in many cancer types [63, 64]. However, growing heterogeneity in intratumoral neoantigens leads to increasing heterogeneity in TILs against tumor cells and in the immune microenvironment [65–67]. A study found liver cancer evolved from different liver diseases may have a distinctive T cell receptor (TCR) repertoire [68]. Consequently, the T cell repertoire coevolves with the tumor cell mutations, and gradually manifests a landscape distinct from those in adjacent normal tissue [69, 70].

The specificity of infiltrating T cells against tumor cells originates from the T cell receptor. Through TCR sequencing, intratumoral T cell heterogeneity with respect to infiltration status, clonality and TCR repertoire was fully characterized in various tumor types. Both spatial and temporal heterogeneity of the immune composition and TCR repertoire in the tumor microenvironment may be pivotal to the fundamentally different responsiveness and prognosis under immunotherapies, as seen in Table 3. The immune responses of different clusters of infiltrating T cells against a tumor are heterogeneous. In one study, clonality and accumulation of high-frequency clonotypes were higher in CD8 + TILs than those of CD4 + TILs, while a higher amount of TCR repertoire diversity was discovered in CD4 + TILs [71]. The complex architecture inside tumors may further complicate the intratumor TCR heterogeneity [72]. Dynamic evaluation of the temporal heterogeneity of TCR repertoire has also been used to reflect immune status, predict distant metastasis after treatment and indicate prognosis [73–75]. The varied vascular and lymphatic spatial distribution may lead to different accessibility to oxygen and nutrients across different regions that shape the microenvironments holding T cells resulting in differing quantities, functions and reactions to neoantigens [72, 76].

**Table 3:** Representative studies revealing ITH of tumor-infiltrating lymphocytes

Tumor type	References	Heterogeneity type	Main indicators of heterogeneity	Compared region	Relationship with prognosis
NSCLC	[66]	Spatial heterogeneity	Tumor-infiltrating T cells	Ubiquitous and multi-regional tumors	Numbers of expanded ubiquitous or regional intratumoral TCRs are not associated with outcome
LC	[41]	Spatial heterogeneity	CD8 + T cell infiltration	Multi-regional tumors	High clonal neoantigen load and low immune evasion capacity are associated with improved disease-free survival times
Localized LUAD	[72]	Spatial heterogeneity	CD4 + and CD8 + T cells	Centers and margins of tumors	Amount and TCR repertoire ITH of CD4 + and CD8 + TILs in tumor centers and margins are associated with prognosis
Localized LUAD	[65]	Spatial heterogeneity	T cell density and clonality	Multi-regional tumors	ITH in the T cell repertoire is associated with a risk of relapse
Early LUAD	[265]	Spatial heterogeneity	Immune cell atlas	Tumor, adjacent tissue and blood	N/A
ESCC	[266]	Spatial heterogeneity	TCR landscape and PD-L1 expression	Multi-regional tumors, normal tissues and blood samples	High proportion of branch neoantigens is associated with short overall survival
ESCC	[119]	Spatial heterogeneity	T cell clonality	Multi-regional tumors, matched adjacent normal tissue and peripheral blood	N/A
CRC	[267]	Spatial heterogeneity	T cell clones and counts	Tumor and adjacent tissue	N/A
GBM	[84]	Spatial heterogeneity	TIL diversity	Multi-regional tumors	Overall level of the immune response is connected with prognosis
OC	[268]	Spatial heterogeneity	T cell clonality	Multi-regional tumors	Combination of mutational processes and immune properties is associated with prognosis
NPC	[168]	Spatial heterogeneity	T cell clonality	Matched tumor, adjacent normal tissue and peripheral blood	A lower diversity of TCR repertoire in tumors than paired tissues or a low similarity between the paired tissues is associated with a poor prognosis
MEL and CRC	[269]	Spatial heterogeneity	T cell clonality	Multi-regional tumors	N/A
MEL	[166]	Spatial heterogeneity	T cell clonality	Metastases	Homogeneous lesions are associated with response to therapy;
MEL	[270]	Spatial heterogeneity	Single-cell analyses of T cells	Metastases	N/A
HCC	[53]	Spatial heterogeneity	CD8 + T cells infiltration and immune markers	Multifocal tumors	N/A
PC	[105]	Spatial heterogeneity	T cell clonality	Multi-regional tumors and peripheral blood	N/A
RCC	[271]	Spatial heterogeneity	The clonal composition of T cell populations	Multi-regional tumors	N/A
OC	[272]	Spatial heterogeneity	T cell oligoclonal expansion	Metastases	N/A
OC	[273]	Spatial heterogeneity	T cell clonality	Tumor and peripheral blood	N/A
BC	[274]	Spatial heterogeneity	T cell clonality	Tumors and lymph nodes	N/A
NSCLC	[127]	Heterogeneity among different levels of PD-1 expression	Transcriptional and metabolic profile of T cells	Different subsets of CD8 + TILs	Presence of PD-1 T cells is associated with both response and survival in patients treated with PD-1 blockade
Metastatic MEL	[275]	Heterogeneity among different levels of PD-1 expression	T cell clonality	Metastases	N/A
Metastatic MEL	[126]	Heterogeneity among different levels of PD-1 expression	Phenotypic traits of CD8^+^ TILs and TCR clonotype	Metastases	N/A
CC	[125]	Temporal heterogeneity	Circulating TCR repertoire	Peripheral blood samples throughout carcinogenesis	Less clonotypes in TCR repertoire of sentinel lymphatic node is associated with poor prognosis
GC	[67]	Temporal heterogeneity	TCR repertoire	Tissue samples at different pathological stages	An 11-gene module related to TCR repertoire is correlated with the overall survival of GC patients
LC	[73]	Temporal heterogeneity	Circulating TCR repertoire	Pre- and post-treatment peripheral blood samples	Increased diversity and high overlap rate between the pre- and post-treatment TCR repertoires indicated clinical benefit
*NPC*	[74]	Temporal heterogeneity	Circulating TCR repertoire	pairwise pre-treatment and post-treatment peripheral blood samples	Ascending TCR diversity and higher similarity between pre- and post-treatment samples showed better distant metastasis-free survival
RCC	[75]	Temporal heterogeneity	Circulating TCR repertoire	peripheral leukocyte samples before and after surgery	Higher baseline TCRB diversity is associated with better prognosis of in stage IV patients
NSCLC	[276]	Temporal heterogeneity	Circulating TCR repertoire	blood samples before and 6 weeks after immunotherapy, and disease progression	the diversity of TCR repertoire and singletons in the TCRβ pool increased after immunotherapy
LUAD	[277]	Temporal heterogeneity	Circulating TCR repertoire	Peripheral blood samples	Higher baseline circulating TCRB diversity was associated with better prognosis The chemotherapeutic agents for advanced lung cancer do not affect adaptive immune function over the first few treatment cycles
MEL in mouse model	[278]	Temporal heterogeneity	TCR repertoire	tumor, draining lymph node (dLN) and peripheral blood samples	N/A
CRC	[279]	Spatial heterogeneity and temporal heterogeneity	Immunoscore (derived from the CD3 + /CD8 + T cell densities)	Spatiotemporally distinct sites of metastases	High immunoscore is associated with the lowest recurrence risk

*BC* Breast cancer, *CC* Cervical cancer, *CRC* Colorectal cancer, *ESCC* Esophageal squamous cell carcinoma, *GBM* Glioblastoma multiforme, *GC* Gastric cancer, *HCC* Hepatocellular carcinoma, *LC* Lung cancer, *LUAD* lung adenocarcinoma, *MEL* Melanoma, *NPC* Nasopharyngeal carcinoma, *NSCLC* non-small cell lung cancer, *OC* Ovarian cancer, *PC* Pancreatic cancer, *RCC* Renal cell carcinomas

The expression of different immunologic elements has long been associated with the prognosis of cancer patients [77–79]. With high TMB and ensuing immune cell infiltration, MSI tumors fall into the type 1 microenvironment according to the category proposed by O'Donnell et al. [80]. As for these tumors, ITH of IFN-γ and TIL may be a pivotal factor leading to resistance against immunotherapy.

### dMMR/MSI facilitates immunotherapy through a pre-existing immunoreactive microenvironment

**Fig. 3:**
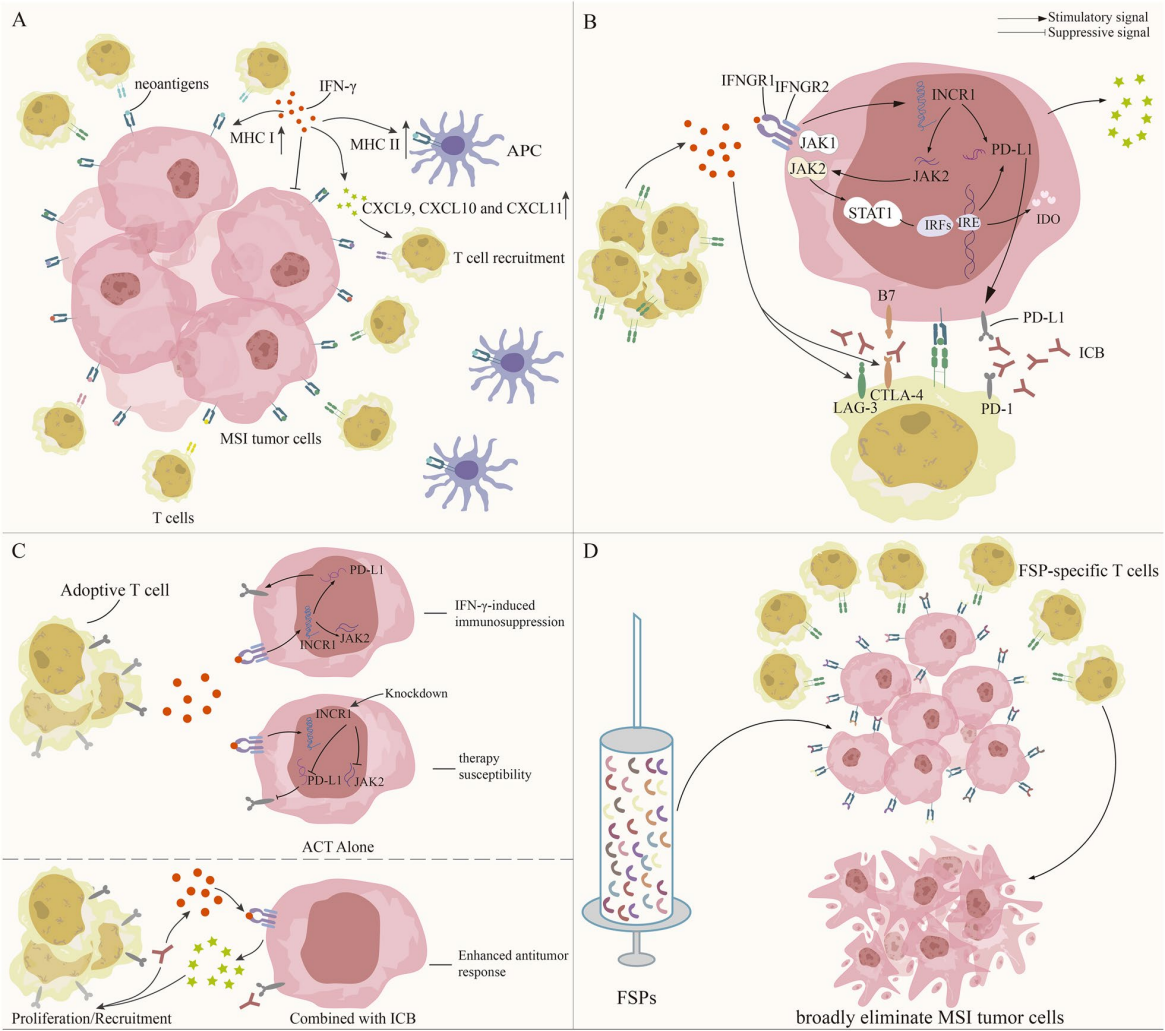
dMMR/MSI facilitates immunotherapy through a pre-existing immunoreactive microenvironment.** a** dMMR/MSI facilitates immunotherapy through: upregulation of IFN-γ signaling; upregulation of MHCI/II and CXCL9/10/11; recruitment of immune cells; direct antiproliferative and pro-apoptotic effects of IFN-γ. **b** IFN-γ induces the expression of immune checkpoints including PD-L1, CTLA-4, LAG-3 and IDO, providing targets for ICB. **c** Silencing IFN-γ signaling to weaken PD-1-PD-L1 interactions helps improve potency of ACT monotherapy. While ICB could improve therapeutic efficacy of ACT through functional IFN-γ signaling. **d** Vaccination with dMMR/MSI-induced antigens could eliminate dMMR/MSI tumor cells and prevent outgrowth of undetected dMMR/MSI subclones

In a recent meta-analysis covering 14 studies, immune checkpoint inhibitors showed encouraging potential in multiple cancer types with dMMR/MSI [81, 82]. While combining Nivolumab with CTLA-4 blockade Ipilimumab exhibits a robust response and improved efficacy [83]. Many other studies have also demonstrated the positive value of dMMR/MSI for immunotherapy, as shown in Table 4. To explore the underlying mechanism, first we need to understand the foundation of effective immunotherapy, which includes: effective antigen presentation by antigen-presenting cells (APC), followed by continuous activation and infiltration of T cells to construct a positive immune microenvironment. In cancer patients without treatment, CD8 + TILs specific to ubiquitously expressed tumor antigens manifest as a dysfunctional phenotype [66]. Immunotherapy triggers the reactivation of the immune system, giving it the ability to identify and react to neoantigens and revitalizing the cytotoxic effect of the pre-existing TIL clonalities [65, 84, 85]. Another premise is sufficient IFN-γ production and responsive IFN-γ signaling. Through this IFN-γ subsequently induces an anti-tumor immune response through: (1) upregulation of antigen processing molecules, MHCI/II and antiangiogenic chemokines (2) recruitment of T cells and other immune cells (3) direct antiproliferative and pro-apoptotic effects [86, 87]. As for ICB, an additional condition is the upregulation of the target immune checkpoint. Continuous IFN-γ exposure induces upregulation of immune checkpoints including PD-L1, CTLA-4, IDO and LAG-3 [87–91], of which the immunosuppressive effect is abrogated and only positive factors come into play in the context of ICB therapy (Fig. 3).

**Table 4:** Biomarkers predicting better response to immunotherapy

Biomarker status	Tumor type	N	Immunotherapy	OS (rate)	PFS (rate)	ORR/Impact	References
dMMR/MSI	CRC, non-CRC (ampullary or cholangiocarcinoma, endometrial, small bowel, gastric)	11, 9, respectively	Pembrolizumab	40%	~ 5 months (20 weeks): 78%	More responsive	[59]
dMMR/MSI	mCRC	74	Nivolumab	31%	12 months: 50%	Durable response and disease control	[81]
dMMR/MSI	mCRC	119	Nivolumab + Ipilimumab	55%	9 months and 12 months: 76% and 71%, respectively	Durable response and improved efficacy	[83]
dMMR/MSI	Recurrent GBM	21	Nivolumab	NA	NA	Initial and durable response	[114]
dMMR/MSI	Advanced, metastatic MSI-H/dMMR CRC	61 in cohort A, 63 in cohort B	Pembrolizumab	mOS: 31.4 months and NR	mPFS: 2.3 months and 4.1 months	33% (95% CI 21–46%) and 33% (95% CI 22–46%)	[280]
dMMR/MSI	27 types of non-CRC	233	Pembrolizumab	mOS:23.5 months	mPFS: 4.1 months	34.3% (95% CI, 28.3–40.8%)	[281]
dMMR/MSI	CRPC	11	Anti-PD-1/PD-L1	NA	NA	Durable clinical benefit: 45.5%	[282]
dMMR/MSI	mCRC	307	Pembrolizumab	NR	mPFS: 16.5 months	Longer progression-free survival	[283]
dMMR/MSI (loss of h MSH2 and MSH6)	Chemo-resistant urothelial tract cancer	1	Durvalumab	NA	NA	Complete remission	[115]
dMMR/MSI, high TMB (> 37–41 mutations/Mb)	mCRC	22	Pembrolizumab, Nivolumab, Nivolumab/Ipilimumab, Durvalumab/Tremelimumab	NA	mPFS: > 18 months,	Objective response	[95]
dMMR/pMMR, higher percentages of mucin and PD-L1 expression	mCRC	26	Pembrolizumab	NA	NA	Clinical benefit	[195]
Higher TMB	Metastatic melanoma	64	Ipilimumab or Tremelimumab	mOS: 4.4 years	NA	Durable clinical response	[97]
Higher TMB	NSCLC	28/22	Anti-PD-1/PDL1 therapies	NA	mPFS: NR/mPFS: 2.9 months	ORR: 39.3%/ORR: 9.1%	[284]
Higher IFN-γ signature (IFN-γ, STAT1, CXCL9, CXCL10, IDO, MHCII HLA-DRA, LAG-3)	Metastatic melanoma, GC, HNSCC	81,33,40	Pembrolizumab	NA	NA	Higher response rate	[182]
Higher IFN-γ signature (IFN-γ, PD-L1, LAG-3 and CXCL9)	NSCLC	30	Durvalumab	Longer OS	Longer mPFS	Higher response rate	[137]
Higher IFN-γ signature (LAG-3, PD-L1, IDO) and TIL	mCRC	19	Pembrolizumab	NA	NA	Higher response rate	[196]
Higher IFN-γ signature and PD-L1 expression	Urothelial carcinoma	265	Nivolumab	mOS:7 months	NA	ORR:28·4% with PD-L1 expression of 5% or greater, 23·8% with PD-L1 expression of 1% or greater, 16·1% with PD-L1 expression of less than 1%	[134]
Higher PD-L1 expression and TMB	Metastatic urothelial carcinoma	310	Atezolizumab	NA	NA	Significantly improved ORR	[113]
Higher PD-L1 expression and TMB	Solid tumors across 22 types	> 300	Pembrolizumab	NA	Longer PFS	Stronger objective response rate	[285]
PD-L1 positive	SCLC, melanoma or RCC	296	Nivolumab	NA	NA	Complete/partial response	[57]
PD-L1 positive	Melanoma, NSCLC, RCC, CRC, CRPC	41	Nivolumab	NA	NA	Objective response and clinical benefit	[112]
Higher IDO expression and TIL	Advanced melanoma	82	Ipilimumab	NA	NA	Better clinical outcome	[135]
INCR1 knockdown	Mice tumor models	-	CAR-T cell therapy	NA	NA	Enhanced T cell infiltration, significantly reduced tumor growth	[130]

*OS* Overall survival*, PFS* Progression-free survival*, ORR* Overall response rate*, dMMR* Mismatch repair deficient*, MSI* Microsatellite instability*, CRC* Colorectal cancer*, mCRC* Metastatic colorectal cancer, *GBM* Glioblastoma multiforme, *CRPC* Castration-resistant prostate cancer, *NSCLC* Non-small cell lung cancer, *GC* Gastric cancer, *HNSCC* Head and neck squamous cell carcinoma, *SCLC* Small cell lung cancer, *RCC* Renal cell cancer, *Pembrolizumab* PD-1 blockade, *Nivolumab* PD-1 blockade, *Ipilimumab* CTLA-4 blockade, *Durvalumab* PD-L1 blockade, *Tremelimumab* CTLA-4 blockade, *Atezolizumab* PD-L1 blockade, *CAR-T cell* Chimeric antigen receptor-T cells, *NA* Not available, *mOS* Median overall survival, *mPFS* Median progression-free survival

Regardless of origin and type [59], dMMR/MSI tumors are susceptible to immunotherapy owing to: (1) high TMB (2) high TIL in both tumor and tumor-adjacent tissues [59, 92] (3) upregulation of PD-1 and IFN-γ signatures (PD-L1, CTLA-4, LAG-3 and IDO) representing an adaptive resistance to the immunoreactive microenvironment induced by MSI [5, 6]. All these three aspects are positive predictive markers [57, 93–95] of which TMB could be considered as the initiating factor. Both cancers with the strongest response to PD-1 blockade have a high degree of mutation, including lung cancer and melanoma [3, 4, 57, 96]. In addition, TMB significantly contributes to a sustained clinical benefit from CTLA-4 blockade in melanoma [97]. With a high mutation load and increased immunogenicity, dMMR/MSI tumors possess abundant infiltration with activated CTL and Th1. They have high expression of cytotoxic genes encoding IFN-γ, signal transducers and activators of transcription 1 (STAT1), interferon regulatory factor 1 (IRF1) and IL18 [5, 98], and more frequent apoptosis of neoplastic cells attributed to both high TIL and intrinsic genetic instability [99]. Higher TIL grade is shown to be associated with better outcomes in different tumor types, including melanoma and CRC [100–102], and intrinsically linked to the response against immune checkpoint inhibitors [103–105]. Despite enhancing tumor immunogenicity, mutator phenotypes with upregulated immune checkpoints could also favor immune evasion and counterbalance the pre-existing anti-tumor immune microenvironment, particularly given the IFN-γ-induced adaptive response. Nevertheless, upregulated immune checkpoints provide targets for ICB to re-invigorate the immune response. In addition, mutations of KRAS and TP53, although not prevalent in MSI tumors, and respectfully favor tumor proliferation and deregulate DNA repair [106–108], TP53 mutation was found to increase expression of immune checkpoints, effector T cells and IFN-γ signature; furthermore, TP53/KRAS co-mutated subgroup manifested increased expression of PD-L1 [109, 110]. Together they may serve as potential predictive biomarker for immunotherapy. Further, WRN dependency was found to be associated with resistance to immunotherapy, in other words, WRN inhibitor may be synergic with immunotherapy, as it increases the genetic instability, and modulates the neoantigen landscape to enhance immune response [40]. Another underlying mechanism facilitating immunotherapy may be higher microvessel density (MVD) found in dMMR/MSI tumors [92], which enables increased lymphocyte extravasation. However, considering angiogenesis benefits for tumor growth, an in-depth study on MVD and MSI is highly recommended.

#### ICB

ICB is one of the most promising anti-tumor immunotherapies to this day. The two most promising targets are CTLA-4 and the interaction of PD-1 and PD-L1. Upregulation of these immune checkpoints is an adaptive resistance associated with poor prognosis [111] and actually represents a strong pre-existing anti-tumor response, based on which ICB is applied to re-invigorate the immune response [57, 112, 113]. dMMR/MSI has been found to promote ICB efficacy in multiple tumor types, including glioblastoma multiforme [114], urothelial tract cancer [115], melanoma [57, 97], endometrial cancer [59], non-small cell lung cancer (NSCLC) [57, 112] gastric cancer [116] (Table 4).

It is believed that oligoclonal expansion of the TIL repertoire is a symbol of low TCR affinity and T cell exhaustion [117], while an appropriate level of TIL heterogeneity may be the foundation of ICB and ACT [118]. In this scenario, ICB could rejuvenate the TCR repertoire extensively rather than focusing only on several T cell epitopes, resulting in more T cells responding to ubiquitous neoantigens, enhancing overall immune competence in the anti-tumor response and leading to most clinically significant responses [119, 120]. Additionally, CD4 + T cells that stimulate and suppress the immunity of CD8 + T cells coexist in the tumor microenvironment [121]. While Tregs are regarded as suppressive regulators in tumor immunology and a biomarker of poor prognosis [122], they still possess specific reactivity against tumor antigens, facilitating CTLA-4 therapy [123]. Although PD-1 indicates negative regulatory function and exhaustion of peripheral T cells induced by the PD-1 signaling pathway and may contribute to the decreased diversity of T cell repertoire [124, 125], CD8 + T cells may function efficiently after PD-1 immunotherapy [126, 127]. Therefore, even though TILs are considered an immunosuppressive phenotype, they possess substantial capacity to induce a cytotoxic effect against tumor cells and their potential proliferation [121].

Among the various cytokines, IFN-γ is the main factor that induces upregulation of PD-L1 [128]. JAK1/2–STAT1/2/3–IRF1 pathway is the most important signaling cascade that is involved [129]. When IFN-γ binds to its receptors interferon-gamma receptor 1/2 (IFNGR 1/2), it increases the level of IFN-stimulated noncoding RNA 1 (INCR1)—a major regulator of IFN-γ signaling by modulating post-transcriptional JAK expression [130]. The subsequent activation of JAK1/2 leads to phosphorylation and dimerization of the downstream signal transducers and activators of transcription (STATs). Then the downstream transcription factors IRFs bind to their response elements IRF-1 response elements 1/2 (IRE1/2) in the upstream 5′-flanking region of the PD-L1 gene promoter [131] and induce PD-L1 upregulation (Fig. 3). A positive correlation between IRFs and PD-L1 mRNA expression was found in hepatocellular carcinoma (HCC) [131]. Similar to PD-L1, the expression of CTLA-4 in human melanoma cells is also regulated by IFN-γ through the JAK1/2-STAT1-IRF1 pathway [132]. CTLA-4 induces antiproliferation of T cells, Tregs activation and upregulation of IDO [133], playing a negative role in anti-tumor immune response. Therefore, anti-CTLA-4 therapy is utilized to increase the ratio of effector T cells to Tregs [87], and, in turn, upregulate IFN-γ production. Higher expression of PD-L1 and IDO predicts a superior response to PD-1 blockade and CTLA-4 blockade (ipilimumab), respectively [57, 134, 135], emphasizing the role of IFN-γ-induced IDO in immune checkpoint blockade therapy. Additionally, IFN-γ can induce MHCII expression, which is correlated with multiple important prognostic pathways and better overall survival rate [58]. In melanoma, MHCII expression is a predictor for anti-PD-1 and anti-PD-L1 response [136]. Altogether, high expression of IFN-γ signaling indicates long-term benefits from ICB [89, 116, 137]. In line with the relationship between PD-L1, CTLA-4, IDO and immunotherapy discussed herein, targeting LAG-3 strongly stimulates CD8 + T cell infiltration and IFN-γ secretion [138, 139], suggesting the possibility of an alternative immunotherapy. Interestingly, blockade of a single immune checkpoint could lead to upregulation of others [140]. For example, inhibition of LAG-3 improves the efficacy of PD-1 blockade in several mouse cancer models [141–144], indicating the better efficacy of combinatorial ICB.

#### ACT

Efficacy of targeting a ubiquitous tumor antigen in adoptive cell therapy has been demonstrated [145]. Specific TCR-transduced T cells are clinically effective in treating patients with metastatic synovial sarcoma [7], while exploiting TILs to recognize multiple tumor neoantigens is effective in single-patient studies on several tumors [70]. Targeting several tumor antigens is an ideal scenario, which circumvents tumor escape mechanisms such as tumor heterogeneity and constructs a focused TIL repertoire against tumor cells [146].

However, the bottleneck of ACT is unable to address T cell migration and abnormal function at tumor sites. A recent study showed that PD-1 expression on transferred T cells could be induced by tumor environment [147], indicating that downregulation of immunosuppressive factors and silencing IFN-γ signaling to weaken PD-1-PD-L1 interactions may help improve potency. INCR1 knockdown cells are more susceptible to cytotoxic T cell-mediated death compared to controlled cells [130]. However, PD-1 blockade could improve therapeutic efficacy of ACT by enhancing T cell proliferation of T cells and upregulating IFN-γ [147, 148]. Importantly, functional IFN-γ signaling could induce chemokine (C-X-C motif) ligand 10 (CXCL10) to recruit more activated T cells and trigger a positive feedback loop [147] (Fig. 3). In addition, PD-1 blockade could increase the activation and proliferation of CAR-T cells in vitro and regress tumor growth in vivo through enhancing their anti-tumor effect and reducing myeloid-derived suppressor cells at tumor sites [149]. Noteworthy, a recent study also revealed that recurrent melanoma after ACT treatment exhibited high expression of IFN-γ signaling (PD-1, PD-L1, CTLA-4, though the picture was heterogeneous), which provided tractable targets for salvage immunotherapy, and indeed allowed for effective ICB [150]. As mentioned, IFN-γ plays an intricate role in ACT. ACT treatment outcomes are different when combined with other therapies due to the heterogeneity of IFN-γ signaling.

#### Vaccination with dMMR/MSI-induced antigens

MMR-deficient subclones progress to manifest dMMR/MSI cancer lesions despite strong immunogenicity and immune surveillance due to upregulation of immune checkpoints and mutations favoring immune evasion. ICB remarkably benefits outcomes of dMMR/MSI tumors; in non-responders, combined with other immune-supportive approaches, it is expected to turn “cold” tumors into “hot” ones and improve the response rate. dMMR/MSI triggers frequent generation of frameshift mutations and gives rise to highly immunogenic frameshift-derived peptides (FSP), which contain multiple immunologically relevant neoepitopes [151]. These neoantigens are tumor-specific and shared by most MSI tumors [152]. A vaccine based on these neoantigens could be designed to prevent outgrowth of undetected dMMR/MSI subclones in pMMR tumors. A clinical Phase I/IIa trial found three commonly mutated FSPs (derived from genes AIM2, HT001 and TAF1B (NCT01461148), of which 98.5% of all MSI CRCs harbor at least one mutation [152]. Theoretically, immune response directed against FSPs can be induced in the majority of MSI CRCs, and the study results confirmed that this FSP vaccination was well tolerated and consistently induced immune responses [153]. The latest research analyzed 320 MSI tumors and selected 209 FSPs to generate a vaccine referred to as Nous-209. The vaccine induced IFN-γ + FSP-specific T cells in vaccinated mice and exhibited strong immunogenicity [154]. Its safety, tolerability and immunogenicity are currently under clinical evaluation in mCRC, gastric and gastro-esophageal cancer patients in combination with Pembrolizumab (NCT04041310) (Table 5). Vaccination with frameshift-derived neoantigen-loaded DC is also under investigation in MSI CRCs and persons who are known to harbor germline MMR gene mutation but without diseases yet (NCT01885702). Despite the therapeutic implications for MSI tumors, this trial could also explore the preventive significance of FSP vaccine for people with MMR mutations. Of note, a vaccine targeting these FSP antigens could broadly eliminate dMMR/MSI tumor cells despite the ITH and rapid tumor evolution, since these mutations are driver events at early stage of tumorigenesis [155, 156] (Fig. 3). Moreover, an IDO-derived peptide vaccine activates IDO-specific T cells which recognize and kill both tumor cells and immunosuppressive dendritic cells in vitro, significantly improving overall survival in III/IV NSCLC patients [157]. As combination therapy may have a synergistic effect due to distinct mechanisms of action, clinical trials are also underway to combine IDO and PD-L1 peptide vaccine with PD-1 blockade to treat metastatic melanoma (NCT03047928). Vaccines based on other upregulated antigens in dMMR/MSI tumors warrant further investigation.

**Table 5:** Ongoing clinical trials investigating immunotherapy in dMMR/MSI tumors

Study group	Trial design	Phase	Current status	NCT number
MSI GIC	Immunotherapy during the perioperative treatment stage	–	Not yet recruiting	NCT04640103
MSI mCRC	At least one administration of PD-1 blockade	–	Recruiting	NCT04612309
MSI locally advanced RC	PD-1 blockade + neoadjuvant chemoradiotherapy (capecitabine plus irinotecan)	II	Not yet recruiting	NCT04411524
MSI mCRC	Avelumab in the 2^nd^ line versus standard chemotherapy ± targeted therapy	II	Recruiting	NCT03186326
MSI mCRC	Modified mFOLFOX6/bevacizumab plus atezolizumab versus single agent atezolizumab	III	Recruiting	NCT02997228
MSI CRC	Nivolumab + Ipilimumab + Radiation therapy	II	Recruiting	NCT03104439
MSI NSCLC, SCLC, UC, HNSCC, MCC, melanoma, RCC, GC, cervical cancer, HCC, CRC	PD-1/PD-L1 blockade + N-803	IIb	Recruiting	NCT03228667
MSI mCRC, READ, other metastatic solid tumors	PD-L1 blockade + TGFbetaRII fusion protein (M7824)	Ib/II	Recruiting	NCT03436563
mCRC	Vaccination with frameshift-derived neoantigen-loaded DC	I/II	Active, not recruiting	NCT01885702
MSI solid tumors	Nivolumab + Relatlimab	II	Recruiting	NCT03607890
MSI localized oesogastric-gastric cancer	Neoadjuvant nivolumab + ipilimumab	II	Recruiting	NCT04006262
MSI advanced solid tumors	FT500 + Nivolumab + Pembrolizumab + Atezolizumab +	I	Recruiting	NCT03841110
Advanced GIC	Pembrolizumab + Wnt inhibitor CGX1321	I	Recruiting	NCT02675946
Advanced dMMR/MSI CRCs	Ipilimumab, nivolumab, oxaliplatin, leucovorin, fluorouracil, irinotecan, bevacizumab, cetuximab	III	Recruiting	NCT04008030
Advanced cancers	NBTXR3 + radiotherapy + PD-1 blockade	I	Recruiting	NCT03589339
dMMR/MSI locally advanced CRCs	Toripalimab + chemoradiotherapy	II	Not yet recruiting	NCT04301557
dMMR/MSI locally advanced or mCRCs	IBI310 + sintilimab	II	Active, not recruiting	NCT04258111
dMMR/MSI CRC, GC and gastro-esophageal junction (G-E junction) tumors	Nous-209 Genetic Vaccine	I	Active, not recruiting	NCT04041310
dMMR/MSI locally advanced RC	Sintilimab ± chemoradiotherapy	II/III	Recruiting	NCT04304209
dMMR/MSI EC, CRC, GC	Neoadjuvant Pembrolizumab	II	Not yet recruiting	NCT04795661
Locally advanced dMMR/MSI CRC	Camrelizumab + Apatinib	II	Recruiting	NCT04715633
dMMR/MSI CRC	Neoadjuvant *Toripalimab* ± Celecoxib	I/II	Recruiting	NCT03926338
dMMR/MSI distal RC	Evaluate the effect and safety of watch and wait in patients accessed pCR after PD-1 monoclonal antibody therapy	NA	Recruiting	NCT04643041
MSI resectable GC/GEJC	Neoadjuvant/definitive treatment of Tremelimumab and Durvalumab	II	Recruiting	NCT04817826
Recurrent and metastatic MSI and non-MSI CRC	Ipilimumab, Nivolumab, Daratumumab, LAG-3 blockade	II	Active, not recruiting	NCT02060188
dMMR/MSI solid tumors	N803 + PD-1/PD-L1 blockade	IIb	Active, not recruiting	NCT03228667
Metastatic/locally advanced/unresectable dMMR/MSI solid tumors	Pembrolizumab + Pevonedistat	I/II	Recruiting	NCT04800627
dMMR/MSI locally advanced *READ*	Neoadjuvant Nivolumab + Ipilimumab + short-course radiation	II	Recruiting	NCT04751370
dMMR/MSI locally advanced solid tumors	Neoadjuvant Pembrolizumab	II	Recruiting	NCT04082572
dMMR/MSI mCRC	Third-line AlloStim immunotherapy	II	Not yet recruiting	NCT04444622
Metastatic melanoma	Nivolumab + peptide vaccine consisting of PD-L1 and IDO	I/II	Recruiting	NCT03047928

*GIC* Gastrointestinal cancer, *mCRC* Metastatic colorectal cancer, *RC* Rectal cancer, *NSCLC* Non-small cell lung cancer, *SCLC* Small cell lung cancer, *HNSCC* Head and neck squamous cell carcinoma, *UC* Urothelial cancer, *MCC* Merkel cell carcinoma, *RCC* Renal cell carcinoma, *GC* Gastric cancer, *HCC* Hepatic cell carcinoma, *READ* rectal adenocarcinoma, *GEJC* Gastro-esophageal junction cancer, *EC* endometrial cancer*, Avelumab* PD-L1 blockade, *mFOLFOX6* Fluorouracil plus leucovorin calcium and oxaliplatin, *N-803* Super antagonist of IL-15*, Relatlimab* LAG-3 blockade, *FT500* Induced pluripotent stem cells (iPSC)-derived NK cell cancer immunotherapy, *NBTXR3* Nano tumor radiotherapy sensitizer, *Toripalimab* PD-1 blockade, *IBI310* CTLA-4 blockade, *Sintilimab* PD-1 blockade, *Camrelizumab* PD-1 blockade, *Apatinib* VEGF inhibitor, *Celecoxib* Cyclooxygenase inhibitor, *Daratumumab* MEK inhibitor*, Pevonedistat* NEDD8-activating enzyme*, NA* Not available

When developing vaccines, a suitable vehicle of transmission can greatly enhance the therapeutic effect. Nanoparticles have been the promising vehicle of vaccine. They are endowed with outstanding physiochemical properties, such as high tissue specificity, manageable surface chemistry and big specific surface area [158]. The nanoparticles can be the vehicle of certain bioactive substance such as PD-L1 inhibitory peptide [159], or be developed with certain features to cause damage to tumor cells [160]. A latest review summarizes two main mechanisms that contribute to the anti-tumor effects of immunotherapy based on nanotechnology: one is to elicit an efficient immune response against tumor during tumorigenesis, while the other is to turn the “cold” immune-suppressive tumor microenvironment into a “hot” immune activated [158].

When exploring treatments for tumor, components of TME such as macrophages, fibroblasts or even tumor vasculature and tumor-draining lymph nodes can be targets of nanoparticles [161]. A vaccine was designed to deliver antigenic microparticle, which transformed tumor infiltrated macrophages into a tumor-suppressive M1 phenotype, and activated strong host immune response against tumor [162]. To enhance the specificity of nanoparticles, particular conditions are used to stimulate the function of the materials. A type of supramolecular gold nanorods can be activated by the second near-infrared-window (NIR-II) light. The nanorods are designed to be the vehicle of CRISPR/Cas9, and they can disrupt PD-1 gene expression of the tumor cells and facilitate immunogenic cell death when irradiated by NIR-II laser [163]. Some other nanoparticles can be released from membrane when entering a microenvironment with specific pH. A short interfering RNA named siFGL1 delivered by nanoparticles with hybrid biomimetic membrane can efficiently silence the FGL1 gene, which is triggered by pH [164]. Whether employed independently or in combination with other immunotherapies as adjuvant, these nanomaterials can enhance immune responses and exhibit anti-tumor efficacy [160, 164].

### dMMR/MSI fuels ITH and also correlates with resistance to immunotherapy

Despite improved efficacy in dMMR/MSI tumors, reported response rates to ICB are variable and often < 50% [95]. What differentiates responders from non-responders? As discussed above, intratumor heterogeneity caused by dMMR/MSI can be a determinant factor leading to the unfavorable response and poor prognosis.

#### ITH impairs the quality of TIL response and impedes immunotherapy

**Fig. 4:**
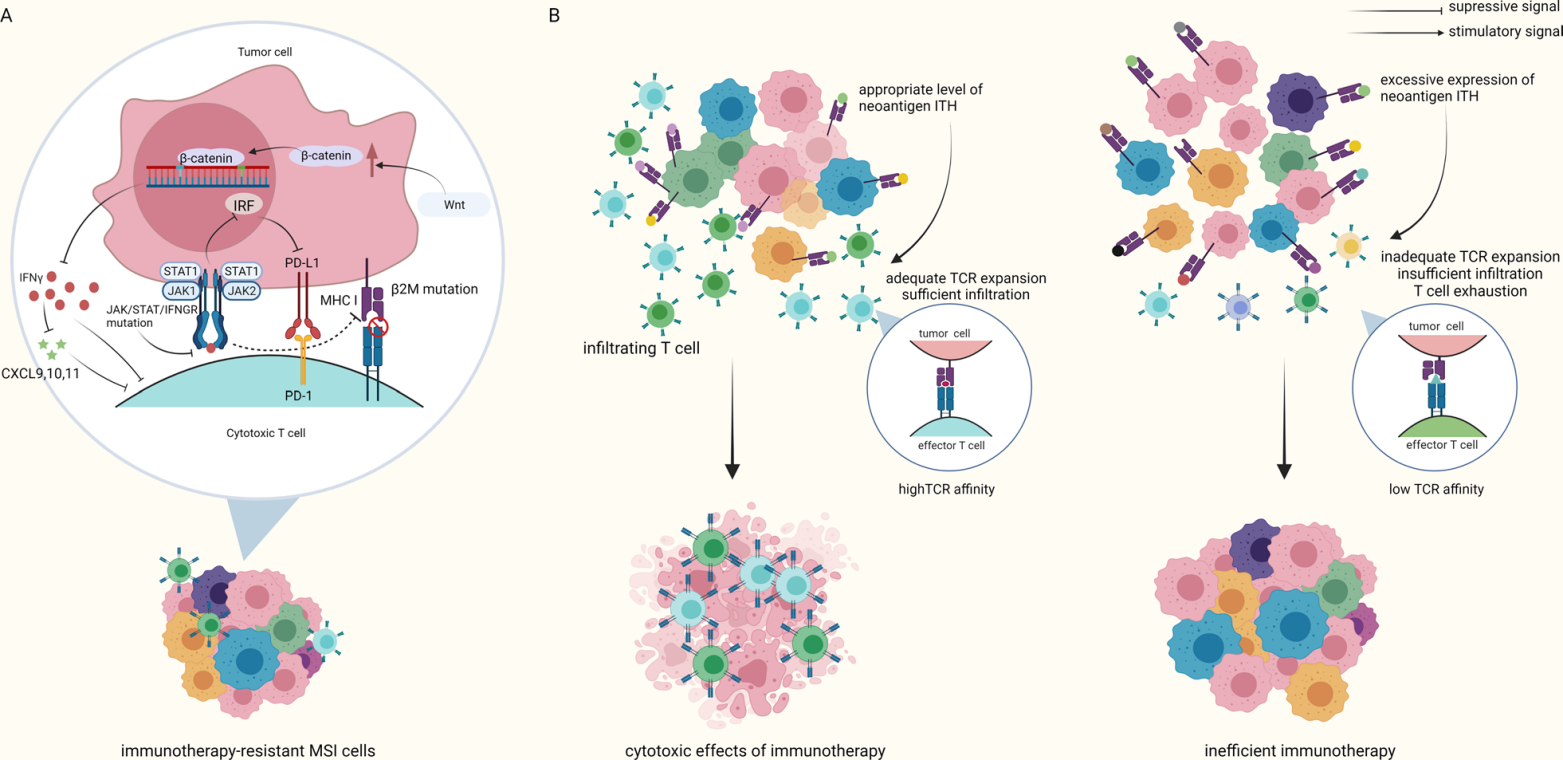
Negative effect of ITH in MSI tumor under immunotherapy. **a** In MSI tumors, hyperactivation of WNT/β-catenin signaling suppresses effector T cells function by reducing IFN-γ. Mutations in JAK and STAT result in impaired IFN-γ signaling and lack of induced MHC class I expression. Moreover, JAK1/2 controls chemoattractant such as CXCL9, CXCL10 and CXCL11, and mutations in JAK1/2 cause lack of downstream T cell infiltration. β2M gene mutations lead to impaired MHC class I function and knockdown of INCR1 decreases PD-L1 expression. Dysfunction of IFN-γ signaling results in lack of PD-L1 expression which leads to PD-L1 blockade out of target, and defective migration of adoptive T cells into tumors in melanoma thereby reducing the efficacy of ICB. **b** Appropriate level of neoantigen ITH leads to adequate TCR expansion, sufficient infiltration and high TCR affinity, which lead to cytotoxic effects of immunotherapy. In the other hand, excessive expression of neoantigen ITH leads to inadequate TCR expansion, insufficient infiltration and T cell exhaustion, which result in inefficient immunotherapy

Although more diversified intratumoral TCRs may be generated in the context of dMMR/MSI, they are not always associated with better clinical outcome [65, 66]. It has long been recognized that tumor progression is accompanied by an increase in tumor mutation load, and the inevitable generation of tumor neoantigens [165]. High ITH is connected to tumor progression and resistant to therapies in many cancer types [47]. Heterogeneity in tumor antigen and immune cells is also significant among melanoma metastases, which leads to different responses to immunotherapy [166]. Excessive expression of subclonal neoantigens may lead to the relatively low expression levels of neoantigens, and T cells may be unable to encounter and activate against those low-frequency neoantigens [167]. Moreover, TCR repertoire diversity is associated with inadequate expansion of TCR clones and deficient infiltration into tumors, which may result from the immunosuppressive state of T cells caused by T cell exhaustion, low TCR affinity, etc. [168, 169]. A higher degree of TCR ITH and consequent clonotypes with low frequencies were revealed in different kinds of tumors and were linked with unfavorable prognosis [65, 170, 171]. Besides, some TILs have lost their functions owing to other dysfunction during the process of immune response. For instance, the tumor antigen TILs previously recognized can be depleted following immunoediting [172, 173], and deprivation of the presenting MHC allele can disrupt antigen presentation [174, 175] (Fig. 4). Therefore, same as above, heterogeneity in the quality of T cell responses, instead of the quantity, may be a determinant factor in anti-tumor response [65].

#### Impact of IFN-γ signaling heterogeneity on immunotherapy

Provided that IFN-γ signaling displays a degree of heterogeneity and its downregulation correlates with an acquired resistance phenotype, alterations of essential components within IFN-γ signaling pathways could modify therapeutic efficacy. Recent studies demonstrate that INCR1 is transcribed as an antisense RNA from the PD-L1/PD-L2 locus and knockdown of INCR1 decreases PD-L1 expression [130]. JAK1/2-deficient cells emerged under/after ICB in patients with advanced melanoma and obtained resistance to PD-L1 blockade, which may result from pre-existing heterogenous subclones or through an adaptive response [9, 176, 177]. JAK loss is possibly correlated with lack of T cell infiltration based on the findings that factors downstream of JAK1/2 controls chemokines with chemoattractant effect on T cells, such as CXCL9, CXCL10 and CXCL11 [113, 178]. Also, high expression of PD-L1 significantly correlates with an objective response to PD-L1 blockade compared to PD-L1 negative patients [112, 113]. Altogether, dysfunction of IFN-γ signaling leads to the lack of PD-L1 expression, resulting in off-target of PD-L1 blockade, and less T cell infiltration for an anti-tumor effect (Fig. 4). Consistent with what’s described above, an interesting study mixed IFN-γ-insensitive tumor cells of melanoma with wild type (WT) tumor cells to mimic ITH. IFN-γ-insensitive cells finally grow out in the context of anti-PD-L1 therapy as a result of (1) failure to activate positive immune response by IFN-γ (2) lack of PD-L1 upregulation as the treatment target (3) immunodepressive microenvironment because of PD-L1 provided by WT. Moreover, IFN-γ could push the tumor further toward the IFN-γ-insensitive cells [179].

In addition, the JAK mutation contributes to the primary resistance to anti-PD-1 therapy in patients with advanced melanoma and colon cancer despite having a high mutation load [59, 96, 180, 181]. In previous studies, copy number alterations (CNAs) and single-nucleotide variants (SNVs) of IFN-γ signaling including loss of IFNGR1/2, JAK1/2, IRF1, as well as amplification of important IFN-γ pathway inhibitors SOCS1 and PIAS4, were found in patients with metastatic melanoma resistant to anti-CTLA-4 therapy. In addition, CXCL10 is reduced compared to the IFN-γ responsive cells [177]. Moreover, the heterogeneity of MHC expression on tumor cells and its lack of coordination with IFN-γ signaling have a significant impact on ICB. In sum, expression of IFN-γ strongly correlates with the response to ICB [182] and has validated in several studies. Deficiency of IFN-γ signaling can weaken the effect of positive immunoregulation in multiple aspects, thereby reducing efficacy of ICB. Diverse subclones carrying heterogenous IFN-γ signaling within tumors have an impact on drug response and should be considered when selecting therapeutic regimens. Given that CTLA-4 blockade leads to increased production of IFN-γ and thereby upregulating PD-L1, combination with PD-L1 blockade could make a better clinical response; and combination with new immune-related targets needs to be studied unremittingly in the future.

Mutations in JAK and STAT result in impaired IFN-γ signaling, lack of induced MHC class I expression, as well as inhibition of the WNT signaling pathway [11, 183]. A study investigating immune evasion in 1,211 CRC patients found that non-responsive dMMR/MSI patients frequently underwent immunoediting through upregulated WNT/β-catenin signaling and complete disruption of key genes in the antigen presentation pathway [7, 8]. High WNT signaling with mutations of β-catenin is inversely correlated with TIL independent of high TMB in melanoma and CRC, thereby reducing the efficacy of ICB [7, 184]. Other studies found that hyperactivation of WNT/β-catenin signaling suppressed effector T cells function by reducing IFN-γ [185] and led to defective migration of adoptive CD8 + T cells into tumors in melanoma [186]. This indicates that WNT signaling inhibitors may reverse immune evasion to facilitate immunotherapy. Approximately 30% of dMMR/MSI CRC display gene alterations of β2 microglobulin (β2M) in that the β2M gene harbors four coding microsatellites (cMS) [152]. β2M gene mutations lead to impaired MHC class I function, defective recognition and presentation of neoantigens which render the immune evasion from immunotherapy [176, 187, 188]. Altogether, mutations of IFN-γ signaling, WNT/β-catenin signaling and antigen presentation machinery, followed by resistance to T cell-induced death could all trace back to dMMR/MSI-induced heterogeneity (Table 6) (Fig. 4). Although high TMB is discussed as a positive predictor of immunotherapy, the quality of mutations to generate a robust T cell response may outweigh the quantity.

**Table 6:** Underlying mechanisms of resistance to immunotherapy

Findings	Tumor type	N	Immunotherapy	Impact	References
LOH in β2M	Metastatic melanoma	160	Ipilimumab, Pembrolizumab	No response	[188]
Deficient IFN-γ pathway genes (IFNGR1, IRF1, JAK2 and IFNGR2)	Melanoma	16	Ipilimumab	No response	[177]
Loss-of-function mutations in JAK1/2, inactivation of β2M	Metastatic melanoma	4	Pembrolizumab	Initial response followed by progression	[176]
Gain-of-function mutations in β-catenin	Metastatic melanoma	266	anti-PD-L1/anti-CTLA-4	Absence of T cell infiltration	[184]
Active β-catenin expression	Melanoma model	–	ACT	No response, resistant to memory CD8 + T Cells	[186]
Biallelic losses of β2M and HLA genes, upregulated WNT/β-catenin signaling	CRC	179	–	Absence of T cell infiltration	[7]
Increased Wnt signaling, decreased IFN-γ levels	Melanoma	31	–	Suppression of induction and effector phases of anti-tumor T cell responses	[185]
Loss-of-function mutations in JAK1/2	Melanoma	169	Anti-PD-L1/anti-CTLA-4	Progressive disease	[9]
Loss-of-function mutations in JAK1/2	Metastatic melanoma, CC	39	Anti-PD-1	No response	[180]

*LOH* Loss of heterozygosity, *IFN-γ* Interferon-gamma, *IFNGR1/2* Interferon-gamma receptor 1/2, *IRF1* Interferon regulatory factor 1, *JAK1/2* Janus kinase 1/2, *HLA* Human leukocyte antigen, *CRC* Colorectal cancer*, CC* Colon cancer, *ACT* Adoptive cell transfer

### Status of MMR system and microsatellite exhibits heterogeneity to some extent

In sporadic CRC cases, which arise from epigenomic silencing by hypermethylation of the MMR gene promotor, MMR deficiency may occur during tumor progression and display tumor heterogeneity (Fig. 1). In 100 cases of sporadic colon cancers, discordance was discovered when IHC and PCR-based microsatellite evaluation were performed in two different areas from the same tumor tissue in 8 cases, of which 6 cases presented normal MMR protein expression but exhibited MSI and 2 cases were the opposite [189], indicating the ITH of dMMR/MSI. In addition, cases reported a coexistence of dMMR and pMMR subclones in the primary lesions of mCRC and prostate cancers, but only pMMR/MSS was detected in the metastatic lesions [190, 191]. dMMR/MSI tumors are less likely to metastasize to regional lymph nodes and distant organs [1, 6] because (1) tumor cells with enhanced antigenicity are more likely to be recognized and localized (2) accumulated DNA damage results in decreased cell viability [192, 193]. There are also some studies verifying the heterogeneity of MSI and MMR protein expression [190, 194]. During the treatment, residual pMMR/MSS cells emerge from mixed subclones and foster temporal heterogeneity, resulting in acquired resistance. Therefore, due to the predictive and therapeutic value of dMMR/MSI, early detection of resistance and targeting the minimal resistant subclones is imperative.

### Combined predictive markers are important to guide precise and personalized immunotherapy

dMMR/MSI, TIL and IFN-γ signaling can altogether reflect the response to immunotherapy. However, there is a disparity between response rate and detected biomarker status. Schrock et al. found the optimal cutoff for TMB as 37–41 mutations/Mb, below which the response to anti-PD-1 monotherapy was inferior despite dMMR/MSI status [95]. This number could be lower with combined ICBs [81], suggesting that combined therapy is preferred to monotherapy for dMMR/MSI patients with TMB below the cutoff. Although pMMR/MSS CRCs account for the majority of total number of CRCs and have a very low response rate to ICB [59], recent studies demonstrated that a subgroup of pMMR mCRC patients also obtained clinical remission from ICB due to higher level of IFN signature (PD-L1, LAG-3, IDO) [195, 196]. Some PD-L1 negative patients also responded to ICB [113, 134] probably due to sampling bias as a result of spatial heterogeneity, or other undetected factors. As discussed above, these markers alone do not predict therapeutic efficacy perfectly on an individual basis, but could make up for each other. Of note, all three features display a certain degree of heterogeneity. Thus, combatting heterogeneity using novel detection methods and better identifying patients’ anti-tumor immune capacity is the key to pre-select those most likely to benefit from treatment and spare others from unnecessary side effects (Fig. 1).

#### Detection methods to combat spatial heterogeneity

The optimal treatment is expected to target the trunk of all subclone mutations and subclonal driver events [19]. Therefore, it is indispensable to overcome the spatial heterogeneity and understand the full range of tumor tissues. The key step is accurate assessment, which is supported by a wealth of progressive studies [28, 197]. The conventional detection methods for dMMR/MSI are PCR and IHC. However, detection accuracy is limited by unfaithful Taq polymerase, limited panel numbers, the necessity for matched normal tissues and experience-dependent IHC [28]. Next-generation sequencing (NGS) allows for comprehensive investigations of multiple microsatellite loci simultaneously. MSI detected by PCR and 592-gene NGS was compared across 26 cancer types and a cutoff of ≥ 46 altered loci was found to classify samples as MSI [198], indicating that MSI-NGS is valid across cancer types and not limited by normal tissue acquisition. Additionally, tools based on NGS including mSing [199], MSIsensor [200], MSIplus [201] and MANTIS [202] have significantly improved sensitivity and specificity.

Several breakthroughs have been made with single-cell sequencing. Tumor cell diversity is analyzed by flow cytometry through a single-cell suspension which fully represents an intact tumor, providing the highest resolution to determine the true number of heterogenous subclones and characterize them without aggregating the information from multiple cells [203, 204]. Among all technologies, transcriptome analysis—single-cell RNA sequencing (scRNA-Seq) is the most advanced [203]. scRNA-Seq sheds light on the tumor immune microenvironment by showing the proportions of TILs. In mCRC samples, proportions of CD8 + T cells, Th1/2 cells and memory T cells were lower, and approximately 81.94% (118/144) of the genes related to WNT signaling were upregulated [205]. Patients with large B cell lymphoma who achieved complete response or remission showed improvement of memory T cells in scRNA-Seq of CAR-T cells [206]. Furthermore, scRNA-Seq identified TILs with high heterogeneity in Osteosarcoma (OS) and high expression of LAG-3 and TIGIT (T cell Immunoreceptor with Ig and ITIM domains) on CD8^+^ T cells, identifying new therapeutic targets for OS [207]. scRNA-Seq could also offer TCR sequence information and provides insight into TCR rearrangements at the single-cell level, unfolding dynamic responses to immunotherapy including vaccine and ICB [208]. TCR sequencing has been widely used and has helped probe into the dynamic combinations of T cell subsets and the spatial heterogeneity of TILs [84, 209, 210]. Single-cell sequencing has identified the heterogeneous expression of IFN-γ-related genes including MHCII in single cells, of which higher expression drives patients’ responsiveness to PD-1 blockade based on longitudinal scRNA-Seq [58, 211]. Enrichment of 227 IFN-γ-dependent transcripts including PD-L1 and IDO was also identified across multiple tumors and could be utilized to stratify immunotherapy response [212]. Mitra et al. found that single-cell analysis of a targeted transcriptome which predicted drug responses for individual cells was able to predict the response to a proteasome inhibitor when combined with machine learning in multiple myeloma [213]. Conceivably, it could also apply to immunotherapy based on correlative transcriptome signatures. Finally, simultaneous triple omics sequencing could reveal complex interplays within genetic, epigenetic and transcriptomic levels and provide the most complete maps of tumor cell subpopulations to guide treatment options [16].

The above discussion prompted us to quantify ITH and stratify patients by classifying potential responses to immunotherapy using combined biomarkers. Studies have classified immune status of tumors into several subtypes to support decision making and facilitate response prediction, based on TIL, IFN-γ signaling signatures and immune checkpoints expression [77, 214, 215]. Future studies should consider including multiple biomarkers to optimize this stratification method.

#### Real-time monitoring: combat temporal heterogeneity

Due to the temporal heterogeneity during natural tumor progressing and therapeutic interventions, it is important to achieve real-time monitoring in a minimally invasive way and promptly adjust therapeutic regimens. Longitudinal analysis of tumor-derived genetic materials including CTCs and ctDNA extracted from patients’ blood has achieved promising progress across several types of solid tumors [216–219]. These materials display all the alterations present in the tumor and the metastasis, which help eliminate false results caused by spatial heterogeneity. ctDNA analysis by liquid biopsy (blood test) is feasible and has been found to be sensitive and specific in various cancer types [220–222]. Studies showed that ctDNA identified genomic profiling highly consistently with and beyond the findings of tissue biopsy [223–228]. In 433 metastatic prostate cancer cases, dMMR identification using ctDNA was highly concordant with IHC and PCR of tumor tissue. Subclonal diversity and β-catenin activation were detected with sensitivity as well [229]. Detection of MSI using ctDNA with NGS in CRC was better than PCR and demonstrated high overall accuracy in pan-cancer [230]. Additionally, an initial peak following by a rapid decrease in ctDNA level indicates an early response for ACT, which in turn allows for early identification of those at risk of poor response and treatment optimization [206, 231]. Analysis of CTC also enables real-time monitoring and provides insight into the genomic profiling [232]. High expression of PD-L1 on CTC at baseline may be predictive to screen patients for PD-1/PD-L1 blockade and reduction of total CTC through longitudinal monitoring indicated a good response [233, 234]. Adjuvant PD-1/PD-L1 blockade deserves evaluation in patients whose PD-L1 ( +) CTCs are detected after curative treatment [235]. The number of CTCs significantly decreased after NK cell treatment in NSCLC and liver cancer, reflecting the therapeutic efficacy with decent sensitivity [236, 237]. Moreover, overexpression of β-catenin was detected in melanoma CTCs, but not in healthy donor and lacking in patients with complete response to ICB [238]. TMB measured from liquid biopsy was also found to be a predictive biomarker for atezolizumab (anti-PD-L1) in NSCLC, and able to identify patients who would benefit accurately and reproducibly [239]. In aggregate, liquid biopsy is a highly sensitive and informative method that can overcome ITH to identify low-frequency alterations and enable early detection of resistance or relapse.

Moreover, imaging techniques also allow for repeated response measurements during treatment, enabling visualization of ITH. Positron-emission tomography (PET) imaging with ^89^Zr-atezolizumab (anti-PD-L1) in NSCLC, bladder and triple-negative breast cancer showed that tracer uptake was heterogenous and corresponded to PD-L1 and IFN-γ signaling levels at sites, appearing to be a strong predictor of atezolizumab response [240]. Radiolabeled [^111^In] PD-L1-mAb and near-infrared dye conjugated NIR-PD-L1-mAb also demonstrably detected graded levels of PD-L1 expression with high specificity using SPECT/CT imaging [241, 242]. Transitioning these detective methods to combat ITH from the bench to bedside and evaluate and monitor patients’ potential benefits from immunotherapy is an enormous challenge that requires more clinical studies.

## Conclusion

Immunotherapy has led to unprecedented long-lasting anti-tumor activity in cancer patients. Currently, clinicians utilize MSI evaluation and other methods, such as IHC of PD-L1, to distinguish those most likely to benefit. However, there are quite a few dMMR/MSI patients who do not respond to immunotherapy as expected. In this review, we explored factors facilitating or impeding immunotherapy from a novel perspective—complex interplay of MSI and ITH. It is commonly believed, and also true, that dMMR/MSI generates subclones with heterogenous genotypes and neoantigens, which stimulate anti-tumor response through higher TIL grade and expression of IFN-γ-related genes. The premises of effective immunotherapy—continuous activation and infiltration of T cells, sufficient IFN-γ production and responsive IFN-γ signaling—are satisfied in this scenario. Nonetheless, non-responders may suffer from the two-sided effects of dMMR/MSI due to a greater tendency for mutations in key elements involved in anti-tumor immunity. Additionally, excessive expression of diversified subclonal neoantigens may lead to relatively low expression of each neoantigen, resulting in inadequate expansion of TCR clones, subsequent T cell exhaustion and insufficient infiltration. Therefore, the subject boils down to one point: the quality of ITH outweighs the quantity.

To better identify patients’ anti-tumor immune capacity and guide individualized immunotherapy, single-cell sequencing uncovers the heterogenous pictures of tumor at the highest resolution, while liquid biopsy achieves real-time monitoring and enables early detection of resistance. Other investigative methods combined with imaging techniques provide multiple directions of future research. The advantage of a dMMR/MSI tumor is the pre-existing immunoreactive microenvironment. To promote and sustain immune activation, immunotherapy needs to be combined with targeted therapies to bypass defects in IFN-γ signaling and antigen presentation machinery, and to inhibit upregulated oncogenic signaling pathways. Many related clinical trials in dMMR/MSI tumors are ongoing, as summarized in Table 5. Moreover, it is important to note that heterogeneity of the MMR system and microsatellite status may cover up the true potency to respond to immunotherapy. Large prospective studies are needed to identify the rate of ITH of dMMR/MSI with accurate detection methods.

## Data Availability

Not applicable.

## Abbreviations

ACT: Adoptive cell transfer
CAR-T: Chimeric antigen receptor-T cells
cMS: Coding microsatellites
CNA: Copy-number alteration
ctDNA: Circulating tumor DNA
CTC: Circulating tumor cells
CTLA-4: Cytotoxic T lymphocyte-associated antigen-4
CXCL: Chemokine (C-X-C motif) ligand
dMMR: Deficient mismatch repair
FSP: Frameshift-derived peptides
HCC: Hepatocellular carcinoma
ICB: Immune checkpoint blockade
IDO: Indolamine-2,3-dioxygenase
IFN-γ: Type II interferon
IFNGR 1/2: Interferon-gamma receptor 1/2
INCR1: IFN-stimulated noncoding RNA 1
IRE: IRF-1 response elements
IRF: Interferon regulatory factor 1
ITH: Intratumor heterogeneity
LAG-3: Lymphocyte activation gene-3
mCRC: Metastatic colorectal cancer
MHC: Major histocompatibility complex
MMR: Mismatch repair
MSI: Microsatellite instability
MSS: Microsatellite stable
MVD: Microvessel density
NAE: NEDD8-activating enzyme
NGS: Next-generation sequencing
NIR-II: The second near-infrared-window
NK: Nature killer
NSCLC: Non-small cell lung cancer
PD-1: Programmed cell death protein 1
PD-L1: Programmed cell death 1 ligand 1
pMMR: Proficient mismatch repair
scRNA-Seq: Single-cell RNA sequencing
SNV: Single-nucleotide variant
STAT: Signal transducers and activators of transcription
TCR: T cell receptor
TIL: Tumor-infiltrating lymphocytes
TMB: Tumor mutation burden
WRN: Werner helicase
β2M: β2 Microglobulin
